# The 2 × 2 Achievement Goals in Sport and Physical Activity Contexts: A Meta-Analytic Test of Context, Gender, Culture, and Socioeconomic Status Differences and Analysis of Motivations, Regulations, Affect, Effort, and Physical Activity Correlates

**DOI:** 10.3390/ejihpe10010015

**Published:** 2019-11-08

**Authors:** Marc Lochbaum, Thaís Zanatta, Zişan Kazak

**Affiliations:** 1 Department of Kinesiology and Sport Management, Texas Tech University, Lubbock, TX 79409-3011, USA; thais.benoit@ttu.edu; 2 Education Academy, Vytautas Magnus University, 44248 Kaunas, Lithuania; 3 Faculty of Sport Sciences, Ege University, Bornova-Izmir 35100, Turkey; f.zisan.kazak@ege.edu.tr

**Keywords:** achievement motivation, approach goal, avoidance goals, correlate outcomes

## Abstract

Approach-avoidance achievement goals are studied extensively in the context of competitive sports and physical activity, including leisure and physical education. Building upon past meta-analyses, the purpose of this quantitative review was to provide basic descriptive data, estimated means for testing of several research questions (i.e., context, gender, culture, and socioeconomic status), and meta-analyzing outcome correlates (i.e., self-determination constructs, affect, effort, and physical activity). A total of 116 studies up to 1 December 2018, met inclusion criteria. These 116 studies, totaling a sample size of 43,133 participants (M sample size = 347.85 + 359.36), from 22 countries with 92.7% of samples, are drawn from participants less than 30 years of mean age. From the 116 unique studies, nearly half (49.6%) were from a sport context and the rest from leisure-time physical activity (PA) (19.4%) and physical education (PE) (31.0%) contexts. A number of different analyses were conducted to examine our research questions. Support was found for several of our research questions: The mastery-approach goal was endorsed more than all the other goals, while sport participants endorsed the performance-approach goal more than PA and PE groups; females endorsed the mastery-avoidance goal more than males; more culturally individualistic countries endorsed the mastery-approach goal; and countries from lower socioeconomic and interdependent countries endorsed the mastery-avoidance goal than higher socioeconomic and independent countries. Concerning, the meta-analyzed correlates, most relationships were hypothesized through the performance-approach goal, and both avoidance goals appeared to be too similar in relationships with the correlates raising theoretical concerns. Overall, the mastery-approach goals had the most meaningful biased corrected effect size values (r_c_) with the outcome correlates, such as relative autonomy (0.47), intrinsic motivation (0.52), effort (0.40), positive affect (0.42), physical activity intent (0.38). Based on the present and past meta-analytic results, the 2 × 2 achievement goals as currently measured was questioned. Future research suggestions included fundamental questionnaire issues, the need for latent profile analysis or other more advanced statistics, and whether the 2 × 2 achievement goal framework is the most appropriate framework in physical activity contexts.

## 1. Introduction

Over twenty years have passed since Elliot et al. [1,2] proposed approach-avoidance achievement goals as an advancement to the much-studied dichotomous achievement goals. Elliot and his colleagues placed their approach-avoidance achievement goals in a framework they termed the Hierarchical Model of Approach and Avoidance Achievement Motivation. In this framework, the achievement goals sit between a number of antecedent categories and commonly accepted achievement motivation outcomes, such as emotions and behaviors. This framework, over the last twenty-plus years, has grown in popularity in the sports, leisure-time physical activity (PA), and physical education (PE) contexts, as evidenced by published meta-analyses in these areas [3,4] and student theses [5,6]. Given the specificity of past meta-analyses (i.e., performance outcomes and achievement goal antecedents), the date of the last search [3,4], and apparent popularity of the these goals, a significant gap still remains in understanding (1) the scope of 2 × 2 achievement goal research, (2) the potential differences in mean values across a number of categorical variables, and (3) the relationship to the achievement goal outcomes other than performance. Thus, the purpose of this review was to fill this gap by addressing a number of research questions along with the hope of providing future research directions.

### 1.1. The 2 × 2 Approach-Avoidance Achievement Goals

Elliot’s approach-avoidance goals stem from the dichotomous achievement goal framework [7]. A recent meta-analysis of 260 studies, concerning the dichotomous goal competitive sports literature [8] and a follow-up meta-analysis with over 700 achievement goal correlate samples [9], show that the dichotomous framework is still much researched. In this dichotomous framework, there are two orientations by which personal competency is judged. Individuals endorsing a task orientation are motivated by personal mastery or improvement. Because of their personal mastery orientation, these individuals gauge their personal competency for a desired behavior by reflecting upon a self-referenced standard of achievement. In contrast, an ego-oriented person strives to attain normative standards of ability. A normative standard of ability is typically defined by winning or beating intended others. Ego-oriented individuals judge their competency on other-referenced standards. 

Initially, Elliot and colleagues [10,11] proposed a trichotomous framework with the mastery, performance-approach, and performance-avoidance goals. These goals were the focus of the Hierarchical Model of Achievement Motivation. Elliot [10] expanded his trichotomous model with bifurcation of the mastery goal into the mastery-approach and mastery-avoidance goals [11]. The dichotomous framework achievement goals of task (mastery) and ego (performance) distinction relate to how competence is defined. The approach-avoidance dimension relates to how competence is valenced. An approach valence indicates a behavior that is initiated by a positive or desirable event or possibility. In contrast, an avoidance valence indicates a behavior that is initiated by a negative or undesirable event or possibility. Thus, approach goals focus on attaining competence. Whereas, avoidance goals focus on avoiding incompetence.

With the 2 × 2 achievement goal framework, competence that is based on the mastery-approach goal is defined by a focus on task-based attainment, such as improving upon one’s past performance in a triathlon. Whereas, competence that is based on the mastery-avoidance goal is defined by a focus on avoiding a worsening of task-based attainment. For instance when bowling, one’s focus with a mastery-avoidance goal would be not to score worse compared to a past performance. For instance, with a personal best of 200, the focus is avoiding scoring less than 200. From the performance goal perspective, the performance-approach goal defines competence based on normative achievements. For instance, a student in a PE class with a performance-approach goal is focused on scoring more basketball points than anyone else in class. With a performance-avoidance goal, an adult exercising in a group class would define competence based on avoiding displays of normative incompetence, thus this individual might indicate a wrist injury to avoid a push-up contest in exercise class.

The measurement of the 2 × 2 goals in the sport, leisure-time PA, and PE domains stemmed from the 12-item Achievement Goal Questionnaire (AGQ) [11], as well as initial work by Cury on the trichotomous framework [12]. Conroy, Elliot, and Hofer [13] published the AGQ-Sport (AGQ-S) that has been widely used in all physical activity settings. Wang, Biddle, and Elliot [14] recognized the need for a PE specific measure. Therefore, they developed a PE specific measure. Last, buried within a two study publication, Stevenson and Lochbaum [15] developed an exercise context version of the 2 × 2 goals from the Elliot and McGregor’s AGQ. Although, the educational literature revised the AGQ [16], no such revision exists in sports, leisure-time PA, or PE literature. Additionally, there are additional approach-avoidance in nature measures, that are not included in this review. For instance, Elliot and his colleagues [17] extended the definitions of achievement goals to task, self, and other crossing with the two approach dimensions for a 3 × 2 achievement goal framework. Most recently, Korn and Elliot [18] proposed and tested the 2 × 2 standpoints model of achievement goals.

Stevenson [5] was the first to quantitatively review Elliot’s goals in the psychology of sport, exercise, and PE domains. Her dissertation, which also examined educational literature, listed nearly 50 studies. Her research demonstrated a difference in the 2 × 2 achievement goal relationships with outcome variables. For instance, Stevenson reported the following meta-analyzed correlations with intrinsic motivation: 0.47 for the mastery-approach goal, 0.15 for the performance-approach goal, 0.01 for the mastery-avoidance goal, and 0.04 for the performance avoidance goal. However, enjoyment as an outcome variable did not show such goal to goal differences: 0.40 for the mastery-approach goal, 0.35 for the performance-approach goal, 0.32 for the mastery-avoidance goal, and 0.27 for the performance avoidance goal. Jean-Noel’s [6] thesis was specific to 17 studies with the approach-avoidance goal and Self-Determination Theory (SDT) constructs with nearly all theoretical relationships being statistically supported, such as the mastery-approach goal being related to relative autonomy, while the other goals were not meaningful in magnitude (meta-analyzed correlation 0.43 mastery-approach goal, 0.09 performance-approach goal, 0.09 mastery-avoidance goal, and −0.05 performance-avoidance goal). Lochbaum and Gottardy [3] reported on 17 studies and quantified the approach-avoidance achievement goals and performance in the sport, leisure-time PA, and PE literatures. Both approach goals were identically related to performance (Hedges’ g = 0.38), while the avoidance goals were not significantly related to performance. Additionally, Lochbaum and Gottardy reported that the performance goal contrast effect size (performance-approach goal minus performance-avoidance goal) was more than twice that of both approach goals in meaningfulness with performance (Hedges’ g = 0.78). Last, Lochbaum and his colleagues [4] meta-analyzed 47 studies specific to Elliot’s [10] hypothesized approach-avoidance goal antecedents, as well as quantifying the intercorrelations among the four goals. Nearly all theoretical antecedent categories to the 2 × 2 achievement goals were supported with small in meaningfulness meta-analyzed correlations. Additionally, the intercorrelations supported the general independence of the four goals.

### 1.2. Purposes and Research Questions

To date, no comprehensive review has been conducted in the sports, PA, and PE domains pulling together all peer-reviewed published research on Elliot’s 2 × 2 achievement goals to test some mean level hypotheses. Specifically, we sought to examine goal level difference; to explore context level differences; hypotheses advanced by Elliot [10] from past avoidance motivation research, concerning greater avoidance goal adoption by females, and from lower socioeconomic individuals in more interdependent cultures; and potential cultural differences in the mastery- and performance-approach goals. Additionally, updated relationships amongst the 2 × 2 achievement goals and achievement outcomes are needed. In summary, we sought to pull together peer-reviewed 2 × 2 quantitatively-based research papers in the competitive sports, leisure-time PA, and PE domains that provided suitable data. To this end, the following six research questions were tested.

#### 1.2.1. Research Question 1

Our first research question concerned the overall pattern of goal endorsement. We hypothesized that the mastery-approach goal will be endorsed significantly and very meaningfully more than the other goals. Our hypothesis is supported by the scale development data in all three domains [13,14,15], and the most recent dichotomous achievement goal meta-analyses reporting the task goal being endorsed more than the ego goal [8,19].

#### 1.2.2. Research Question 2

One main advantage of meta-analytic synthesis is the ability to explore a research question, that has not specifically been tested in the literature. We sought to explore potential context level differences. It seems logical that participants in PE classes will endorse the avoidance goals more than individuals competing in sports and in leisure-time PA, given the compulsory nature of PE, compared to other contexts. If differences exist, then steps could be taken to ensure a more further motivating PE environment. Additionally, it is also logical to expect those participating in sports to endorse the performance approach goal more than PA and PE groups, and the mastery approach goal more than the PE students, given the inherent competitive nature of sports.

#### 1.2.3. Research Question 3

Our third research question concerned sex differences. We hypothesized that females will endorse the performance-avoidance goals more than males. Although, Elliot’s [10] original theories only extended to performance-avoidance goals, we also tested his hypothesis with the mastery-avoidance goal.

#### 1.2.4. Research Question 4

Much research [20] has examined the individualism, more independent, and collectivism, more interdependent. Additionally, Lochbaum and his colleagues [8] tested this hypothesis within the dichotomous achievement goal framework and reported moderate to strong support, that individualistic countries promoted the task goal orientation more so than collectivist countries. Individualism-collectivism hypotheses have been found to hold many core psychological constructs, such as self-concept and attribution and cognitive style [20]. The specific hypothesis tested was countries more culturally individualistic because these countries are more independent, unique, and less group-oriented will endorse the mastery-approach goal more and the performance-approach goal, less so than interdependent countries that are more collectivistic, group focused, and less concerned with the self.

#### 1.2.5. Research Question 5

This research question concerned Elliot’s [10] hypothesized relationship between socioeconomic and interdependent countries, and the performance-avoidance goal. Specifically, the samples from lower socioeconomic and interdependent countries will endorse the performance-avoidance goal more than higher socioeconomic and independent countries, because the lower socioeconomic status potentially highlights increased relational-based achievement goal antecedents, such as a fear of rejection and antecedents to avoidance goal adoption [4]. As with hypothesis 3, we extended this hypothesis based on Elliot’s [10] predictions to the mastery-avoidance goal. Of hypotheses 2-5, only Lochbaum and his colleagues [9] reported correlational support for women endorsing both avoidance goals more than males though the relationships were small. It could be with mean level data, this hypothesis can be explored to a greater extent than Lochbaum and colleagues [9] reported on a few correlate samples.

#### 1.2.6. Research Question 6

Our last research question investigated outcome correlates found in the reviewed literature. The mastery-approach goal was expected to be related positively to outcomes, such as intrinsic motivation, positive affect, and achievement behaviors. The performance-approach goal pattern was also expected to show a positive trend with the outcomes mentioned above, but lower in magnitude than the mastery-approach correlations [4,5,6]. Given the four meta-analytic reviews have reported small to not significant relationships with the avoidance goals and reported correlates [3,4,5,6], we hypothesize a similar pattern to emerge with our outcomes.

## 2. Materials and Methods

### 2.1. Search Strategy and Inclusion Criteria

**Figure 1:**
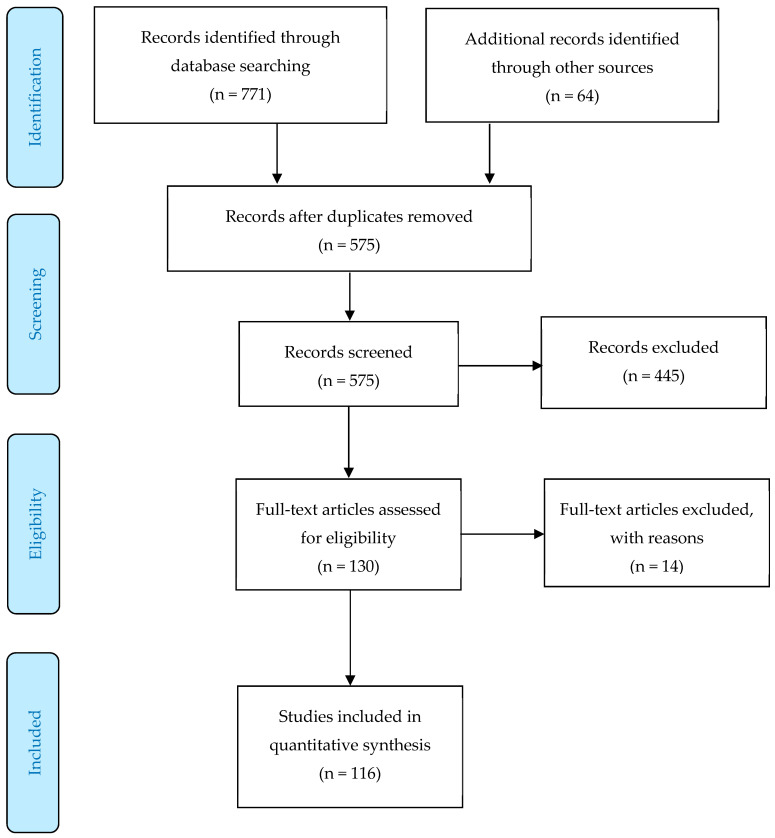
PRISMA flow chart for the identification of the 116 included studies.

The literature search was systematic and comprehensive, based on the PRISMA flowchart [21] components (see Figure 1): Identification, screening, eligibility, and inclusion. The literature search began with the Lochbaum and colleagues’ meta-analyses [3,4]. The screening included electronic databases and search of references from retrieved articles. The electronic database search was conducted in EBSCO with individual databases specific to sport (SPORTDiscus), psychology (PsycINFO), and education (ERIC). To locate the published studies, the following words terms were searched in combination: Mastery-approach goal, mastery-avoidance goal, performance-approach goal, performance-avoidance goal, approach and avoidance goal orientations, sports, competitive sports, physical activity, recreation, leisure-time physical activity, physical education, PE, and exercise. A Supplemental Table is provided detailing the search strategy that started with the year 1999 and ended on 1 December 2018. The protocol for this meta-analysis was not registered.

The articles retained for the purpose of this quantitative review met the following inclusion criteria: (1) Papers must be in any written language that the authors can read with confidence, with or without assistance of a native speaker; (2) papers must be published before 1 December 2018; (3) papers must use original data published in peer-reviewed journals and not repeated in a subsequent publication, and not in theses, book chapters, or conference proceedings that contained data for at least one of the tested hypotheses; (4) the participants must have been in settings such as sports, leisure-time recreational sports, leisure-time exercise, and PE; (5) papers must quantitatively contain one of the 2 × 2 approach-avoidance achievement goals; and (6) measured by a AGQ scale based on 12-items or 3-items per goal.

### 2.2. Data Analysis Procedures

For all the included studies, the following study characteristic data, found in Table 1, were coded: (a) Author name or names, (b) year published, (c) country of study, (d) context, activity, or sport, (e) the approach-avoidance goals found in the study, (f) total sample size, (g) sex makeup of the sample, (h) mean age of participants, and (i) the data extracted. The countries in bold indicated the published manuscript was in the language of that country (e.g., Spain is meant to be written in Spanish). Additionally, bold italics for the mean age of the sample indicated the mean age was estimated with confidence, such as the age range provided. A number of analyses were conducted to test our hypotheses, and provided descriptive characteristics of the included studies. To do so, IBM SPSS version 22 (IBM Corp., 2013) or Comprehensive Meta-Analysis (CMA) version-3 software (version 3.3.070, Biostat, Inc., November 20, 2014) were used.

Prior to testing our hypotheses, IBM SPSS was used to calculate the basic study characteristic statistics. For our mean level hypothesis analyses, the estimate of the means option was chosen for continuous mean data in the CMA program. Means, standard deviations, and sample sizes were inputted for the studies that provided those data. We created categorical moderator variables to test our first three hypotheses. Those moderator variables were goal type, context, and gender sample makeup. The CMA group mixed effects analysis option was chosen for these analyses. Within the mixed effects analysis, the random effects model was used to combine all studies within the chosen subgroup, while the fixed effect model was used to combine subgroups and yield the overall effect. The variance was assumed not to be the same for all subgroups. Thus, variance was computed within the subgroups as opposed to pooled. The mixed effects overall effect output was reported. If the group mixed methods analysis was significant (*p* < 0.05), based on the *Q* between (*Q_b_*) statistic, then 95% confidence intervals (95% CI) were examined for overlap with non-overlapping CI being of most interest, and effect size differences between levels within the moderator variable were calculated with Hedges’ [22] effect size statistic (hereafter noted as *g*). Cohen’s [23] interpretation for computed effect size differences criteria were used with 0.20 as small, 0.50 as medium, 0.80 as large, and 1.30 as very large.

For hypothesis four, IBM SPSS was used to calculate the correlation between the approach goals and the degree of cultural individualism, as well as examine the correlation with socioeconomic status control, using the partial correlation analysis. The International Monetary Fund’s (https://www.imf.org/) World Economic Outlook database was used to find a value to represent the socioeconomic status. In particular, the gross domestic project purchasing power parity (GDP PPP) per capita was used for each study’s year of publication. A few resources were examined determine scores for degree of country individualism such as Eupedia (http://www.eupedia.com/) and Target Maps (https://www.targetmap.com/viewer.aspx?reportId=48440). To test hypothesis five, IBM SPSS was again utilized. Extreme groups were made for country interdependence/independent as the best test of this hypothesis. A univariate analysis of variance (ANOVA) was run with socioeconomic status as the covariate. This analysis was run separately for both avoidance goals.

For all of our outcome correlate analyses, the random effects model option within CMA was used with the mean weight correlation (*r_w_*) as the measure of effect size [24]. Cohen’s [23] criteria were used for interpretation of all correlations: 0.10 to 0.30 as small, 0.30 to 0.50 as medium, and >0.50 as large.

### 2.3. Publication Bias

Publication bias, the publication of hypothesis supportive results, is a concern in a quantitative review. For data run in CMA, the program provides a number of analyses to examine publication bias. The funnel plot [25], the fail-safe N calculation [26], and the ‘trim and fill’ procedure [27] were used. The fail-safe N statistic is interpreted as the number of samples required to change a significant effect size into a non-significant effect size. The greater the value, the more confidence one has that the meta-analyzed result is indeed safe from publication bias. The number of studies per reported study value was used based on the one-tail test. Thus, the larger number of studies per reported study value, the greater the confidence in the effect size being free of publication bias.

Funnel plots were examined to determine if the entered studies were dispersed equally on either side of the overall effect. Symmetry theoretically represents the entered studies, which have captured the essence of all relevant studies. To fix any asymmetry, Duval and Tweedie’s [27] trim and fill analysis was used. Both the number of samples needed and the resultant meta-analyzed effect size is provided in the CMA output. The first author examined each funnel plot and conducted the correction analysis for each reported meta-analyzed correlation. The data points were either filled to the left (i.e., lowering the effect size value) or right (i.e., increasing the effect size value) of the mean, depending upon the where the symmetry was lacking.

### 2.4. Statistical Assumptions of Error

Two primary models are used to determine the statistical assumptions of error. The fixed effects model assumes that all gathered studies share a common effect and differences are a result of within-study error or sampling error. The random effects model assumes both, within study error and between-study variation. Given the extensive variety of studies, the random effects model was chosen when possible within CMA as both, within study error, and between-study variations most likely exist. Lochbaum and his colleagues [4] chose the fixed effects model, however, examination of their heterogeneity values (Table 2, page 74) strongly suggest heterogeneity was present in the majority of their analyses.

Even though it was anticipated that high heterogeneity would be present, heterogeneity was initially analyzed to understand the nature of the data and justify the random effect model choice. A number of statistics exists that measure heterogeneity. For the present investigation, the *I*^2^ statistic was used. The *I*^2^ statistic is the ratio of excess dispersion to total dispersion. As explained by Higgins and colleagues [28,29], *I*^2^ may be interpreted as the overlap of confidence intervals explaining the total variance attributed to the covariates. Higgins and Thompson [29] have provided a tentative classification of *I*^2^ values to help interpret magnitude of the heterogeneity of variance: 25 (low), 50 (medium), and 75 (high).

## 3. Results

### 3.1. Sample Descriptive Summary

Table 1 [13,14,15,30,31,32,33,34,35,36,37,38,39,40,41,42,43,44,45,46,47,48,49,50,51,52,53,54,55,56,57,58,59,60,61,62,63,64,65,66,67,68,69,70,71,72,73,74,75,76,77,78,79,80,81,82,83,84,85,86,87,88,89,90,91,92,93,94,95,96,97,98,99,100,101,102,103,104,105,106,107,108,109,110,111,112,113,114,115,116,117,118,119,120,121,122,123,124,125,126,127,128,129,130,131,132,133,134,135,136,137,138,139,140,141,142] provides a concise description of the located 116 studies fitting study inclusion criteria from 1999 until the search process stopped on December 1, 2018 (see Figure 1 for PRISMA flow chart). All studies except four provided usable mean level data with the additional four providing only correlate data. Table 1 contains author, publication year, study context (sports, PA, and PE), country study completed, participant activity/sport, goals measured, sample size, sex makeup of sample, and mean age of sample. The studies with more than one sample of reported independent data have multiple rows in Table 1. No studies were excluded because of a language barrier. A few authors were contacted for verification of data. The list of excluded studies as a result of insufficient data are available from this study’s first author.

The samples totaled 43,133 participants (M sample 347.85 ± 359.36, range 3 to 2168) originating from 22 countries with the USA (22.5%), Spain (17.8%), and the UK (14.0%) as the most represented countries with distinct samples. The majority (75.0%) of the 116 included studies are from 2010 onward. Of the 116 studies, nearly half (49.6%) came from competitive sports, 31.0% from PE, and 19.4% from PA. Three studies reported mixed sport and PA samples. Within the sport studies, a wide variety of individual and team sports are found in the ‘Activity’ column of Table 1. Both females and males (*n* = 108) were represented in most of the 116 included studies with males only samples being in 13 studies and females only in 6 samples, and not reported in 2. Concerning reported samples’ mean ages, just over half were between 11 and 17 years of age with the average being 18.45 ± 5.56. Only, five studies sampled participants with a mean age greater than 30 years. Two authors, Lochbaum (*N* = 9) and Wang (*N* = 8) within the authors’ research groups contributed the most articles. Last, 10 of the articles were written in Spanish, 3 in Turkish, 1 in Italian, and the rest in English.

### 3.2. Research Question 1 Results

**Figure 2:**
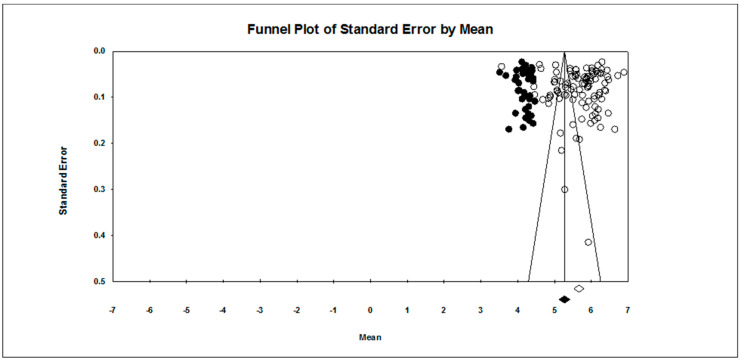
Random effects funnel plot of standard error by mean for the mastery-approach goal on the 7-point response scale samples. Clear circles are observed data, filled in circles are imputed data.

**Figure 3:**
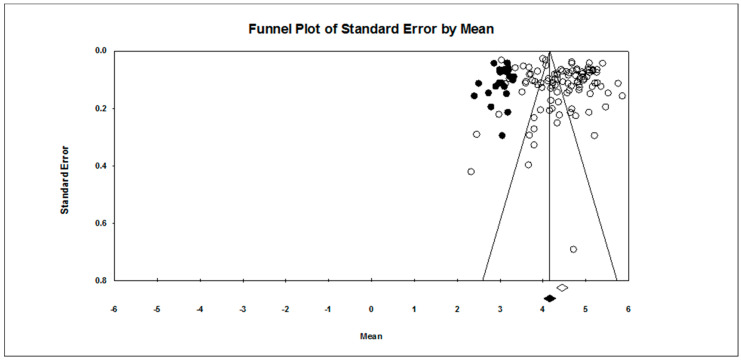
Random effects funnel plot of standard error by mean for the mastery-avoidance goal for the 7-point response scale samples. Clear circles are observed data, filled in circles are imputed data.

**Figure 4:**
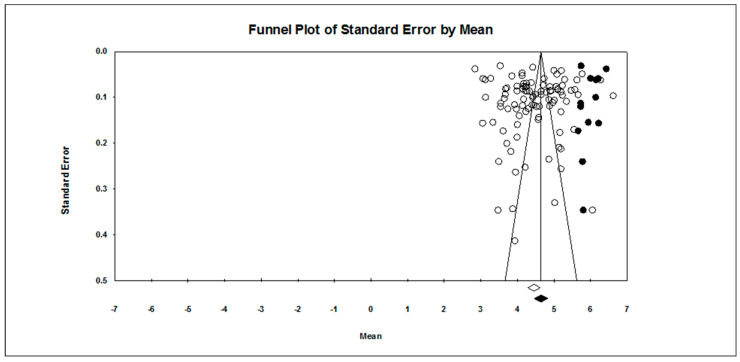
Random effects funnel plot of standard error by mean for the performance-approach goal for the 7-point response scale samples. Clear circles are observed data, filled in circles are imputed data.

**Figure 5:**
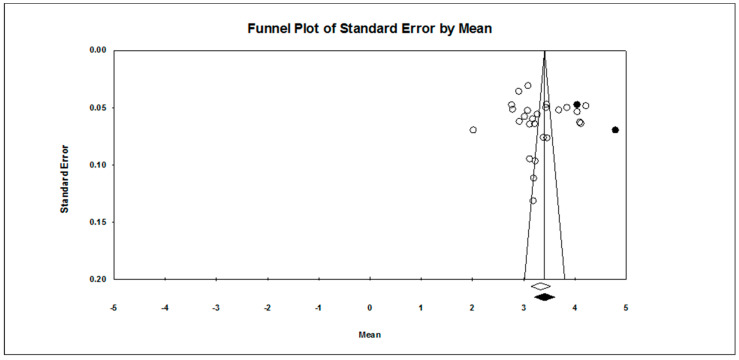
Random effects funnel plot of standard error by mean for the mastery-avoidance goal for the 5-point response scale samples. Clear circles are observed data, filled in circles are imputed data.

**Figure 6:**
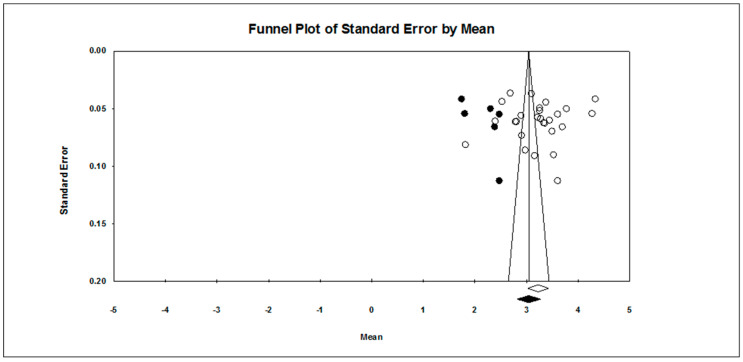
Random effects funnel plot of standard error by mean for the performance-approach goal for the 5-point response scale samples. Clear circles are observed data, filled in circles are imputed data.

**Figure 7:**
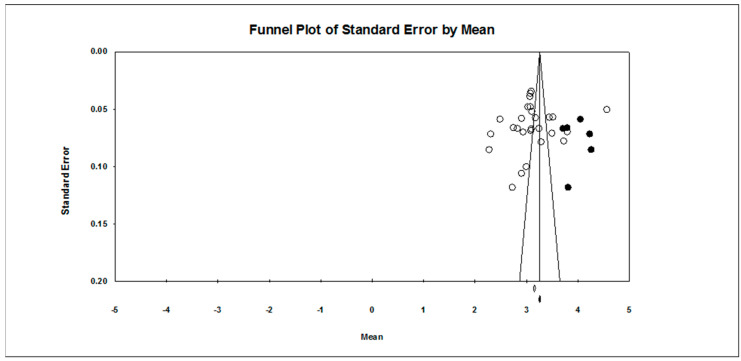
Random effects funnel plot of standard error by mean for the performance-avoidance goal for the 5-point response scale samples. Clear circles are observed data, filled in circles are imputed data.

Our first research question concerned the overall pattern of achievement goal endorsement. As found in Table 2, regardless of scale type, the mastery approach goal is endorsed more than the other three goals hypothesized. The publication bias statistics indicated little bias in the data. Figures for the 7-point scale (Figure 2, mastery-approach goal; Figure 3, mastery-avoidance; Figure 4, performance-approach goal) and 5-point scale (Figure 5, mastery-avoidance goal; Figure 6, performance-approach goal; Figure 7, performance-avoidance goal) of the random effects funnel plots of standard error by mean are presented for each goal, requiring trim and fill. If anything, the approach goals should be adjusted a bit downward, and the avoidance goals adjusted ever so slightly upwards if at all. Certainly, the values in all probability will never have a confidence interval crossing zero given the extremely large missing studies values. Additionally, the use of the random effects model for the remaining analyses, where possible, was justified as all I2 values were high.

To examine the statistical differences across goals, a group mixed effects analysis was conducted. For both scales, the group mixed effects analysis was significant by examining the Qb for both scale types, 7-point scale, Qb(3) = 277.91, *p* < 0.05; 5-point scale, Qb(3) = 66.16, *p* < 0.05. As located in Table 2, the participants regardless of scale endorsed, as hypothesized, the mastery-approach goal significantly more than the other three goals, as verified by examination of the 95% confidence intervals. The mastery approach goal 95% CI lower limit is greater than the 95% CI upper limit of the other goals. The non-overlapping CI held even with the trimmed data analyses. For the 7-point scale only, the performance-approach goal lower limit was higher than the upper limit of the performance-avoidance goal. Thus, the performance goals also differed significantly. The meaningfulness of difference, between the mastery-approach goal and other goals, was very large (g range 1.42 to 2.12) for both scale types. The meaningfulness of difference among the other three goals were nearly zero (g = 0.04, performance-approach/mastery-avoidance 7-point scale) to small (g = 0.20, performance-approach/performance-avoidance 5-point scale; g = 0.21, mastery-avoidance/performance-approach 5-point scale; g = 0.36, mastery-avoidance/performance-avoidance 7-point scale; g = 0.42, performance-approach/performance-avoidance 7-point scale; g = 0.43, mastery-approach/performance-avoidance 5-point scale).

### 3.3. Research Question 2 Results

Our second research question examined whether any differences existed between or among the study contexts. Specifically, we reasoned that participants in PE classes would endorse both avoidance goal more than individuals competing in sports and in leisure-time PA, and those participating in sport and PA would endorse both approach goals more than PE students. To test our thoughts, the group mixed effects analyses were conducted for each goal with each context included in the analysis. The results are presented in Table 3. For the avoidance goals, the group mixed effects analysis was significant for the 7-point performance-avoidance goal, Qb(3) = 8.89, *p* < 0.05, but not for the 5-point performance-avoidance goal. The PE group’s 95% CI lower limit for the significant 7-point scale finding did not overlap with the sport group 95% CI upper limit though did overlap with the PA group’s 95% CI. The meaningfulness of differences were medium between the PE and PA (g = 0.62) and sport (g = 0.71) group. The 5-point mastery-avoidance goal analysis was significant, Qb(3) = 7.73, *p* < 0.05. The sport group endorsed the mastery-avoidance goal more so than the PE (g = 0.49) and PA (g = 1.51) groups and the PE group endorsed the mastery-avoidance goal more than the PA group (g = 0.82). The 7-point mastery-avoidance goal group mixed-effects analysis was not significant.

### 3.4. Research Question 3 Results

Research question 3 examined whether females do indeed endorse the avoidance goals than the males. Most studies had a mix of males and females in the samples. These studies were not included in this analysis. For the 7-point scale data, females (M = 4.66, SE = 0.19, 95% CI = 4.29, 5.03) endorsed the mastery-avoidance goal more than males (M = 4.10, SE = 0.18, 95% CI = 3.75, 4.45), Qb(1) = 4.55, *p* < 0.05. This difference was medium in meaningfulness (g = 0.74). The group mixed effects analysis was not significantly different for the performance-avoidance goal, Qb(1) = 0.03, *p* > 0.05. The 5-point scale was not analyzed as there were few male (*n* = 4) or female (*n* = 1) only studies. In addition to the avoidance goals, we explored approach goal differences. Males significantly (*p* < 0.05) endorsed the performance-approach goal more than females, Qb(1) = 5.04, *p* < 0.05, and the difference was medium in meaningfulness (g = 0.73). Males and females did not differ on the mastery-approach goal, Qb(1) = 0.44, *p* > 0.05.

### 3.5. Research Question 4 Results

Although, the hypotheses were separate for approach and avoidance goals, research question 4 and 5 constructs were tested together, as each country was rated on both socioeconomic status and individualism, and some countries were high in one characteristic but low in another (e.g., Singapore high in socioeconomic status, but low in individualism). Research question 4 tested whether countries more culturally individualistic (independent) endorsed the mastery-approach goal more, and the performance-approach goal less so than countries less individualistic (more interdependent/collectivist). The correlation between individualism and the mastery-approach goal hypothesis from the 5-point scale studies supported this hypothesis with a medium in meaningfulness correlation with, r = 0.33, *p* < 0.05, *n* = 27, and without GDP PPP per capita being controlled (c), rc = 0.31, *p* > 0.05, *n* = 28. Though both were small in meaningfulness, the correlation with GDP PPP per capita being controlled, rc = 0.19, *p* < 0.05, *n* = 102, improved the 7-point scale individualism and mastery-approach goal statistical significance from the non-controlled correlation, r = 0.12, *p* > 0.05, *n* = 105. None of the correlations with the performance-approach goal was significant or even at least small in meaningfulness.

### 3.6. Research Question 5 Results

Research question 5 examined whether samples from lower socioeconomic and interdependent (more collectivist) countries would endorse both avoidance goals more than higher socioeconomic and independent countries. To test this hypothesis, Chile, Costa Rica, Mexico, Singapore, and Taiwan were categorized as low individualism/high collectivism group based on examining individualism estimates by country. Likewise, Australia, Belgium, Canada, France, Sweden, The Netherlands, UK, and USA made up the high individualism/low collectivism group, based on examining individualism estimates by country. A univariate analysis of variance was run with GDP PPP per capita as the covariate.

For the mastery-approach goal 7-point scale analysis, the means were in the hypothesized direction, 4.62 + 0.73, *n* = 13 (low individualism/high collectivism) and 4.32 + 0.70, *n* = 60. The meaningfulness of difference was medium between the two groups, g = 0.42. The covariate was significant, F(1) = 5.62, *p* < 0.05. However, the univariate F test for group was not significant, F(1) = 2.31, *p* > 0.05. Given the covariate was significant, the estimated means were examined. The low individualism/high collectivism group mean was nearly identical without the covariate, 4.63 + 0.68 (95% CI = 4.25, 5.01) whereas the high individualism/low collectivism group mean lowered, 4.14 + 0.68 (95% CI = 4.25, 5.01). The effect size value now was medium in meaningfulness, g = 0.71.

As with the 7-point scale mastery-approach goal analysis, the 5-point scale mastery-avoidance means were in the hypothesized direction, 3.71 + 0.42, *n* = 5 (low individualism/high collectivism) and 3.43 + 0.76, *n* = 8. The meaningfulness of difference was medium between the two groups, g = 0.40. Given the limited samples, the analysis was underpowered. The covariate did not reach traditional statistical significance, F(1) = 4.01, *p* = 0.07. The univariate F test for group was not significant, F(1) = 0.99, *p* > 0.05. Given the limited samples, the estimated means were examined. The means barely changed for the low individualism/high collectivism group, 3.74 + 0.59 (95% CI = 3.16, 4.32) and the high individualism/low collectivism group, 3.41 + 0.58 (95% CI = 2.95, 3.87). The effect size was still medium in meaningfulness, g = 0.52.

For both the scale types, none of the analyses or mean level data supported the differences in the performance-avoidance goal between the two extreme groups.

### 3.7. Research Question 6 Results

Our last research question dealt with achievement goals and outcome variables. We hypothesized that the mastery-approach goal was positively related to outcomes, such as intrinsic motivation, positive affect, and achievement behaviors, and negatively related to motivation and negative affect. The performance-approach goal pattern was expected to show a positive trend with the outcomes mentioned above, but lower in magnitude than the mastery-approach correlations. It is positively related to amotivation and potentially negative affect, and generally small to not significant relationships with the avoidance goals and reported correlates.

To test our hypotheses, a total of 442 correlations were extracted across 11 categories, of which seven fell within SDT. The other categories were positive and negative affect, effort, and physical activity (intent, objective measures, and self-reported). Table 4 contains the categories of studies contributing the outcome correlates. Intrinsic motivation had the most samples (k = 19). The number of correlations per approach-avoidance goal were nearly identical (109 performance-approach, 106 performance-avoidance, 104 mastery-approach, and 103 mastery-avoidance). Of the 11 categories across the four achievement goals, 20 required adjustment of which none which changed more than 0.08 in magnitude (mastery-approach goal and self-reported PA from 0.31 to 0.39). Concerning statistical significance, 12 meta-analyzed correlations were not reliably (*p* < 0.05) different than 0, based on 95% CI. By looking broadly across the correlations by goal and category, we found that the hypotheses concerning the approach goals were generally supported. Whereas, the avoidance goals random effect correlations were not.

All of the mastery-approach goal correlations were significantly different than zero (Z statistic *p* < 0.05). The effect size as found in Table 4 with relative autonomy was large (r_w_ = 0.51), medium with intrinsic motivation (r_w_ = 0.48),extrinsic motivation (r_w_ = 0.36), identified regulation (r_w_ = 0.42), positive affect (r_w_ = 0.42), effort (r_w_ = 0.40), and physical activity intent (r_w_ = 0.38) and self-reported physical activity (r_w_ = 0.31), and small with introjected regulation (r_w_ = 0.28), external regulation (r_w_ = 0.15), amotivation (r_w_ = −0.22), negative affect (r_w_ = −0.12), and objectively measured physical activity (r_w_ = 0.23).

For the mastery-avoidance goal the Z statistic was significantly different than 0 for eight of the 11 correlate categories and all positive in direction. The correlates were medium in meaningfulness for extrinsic motivation (r_w_ = 0.39) and introjected regulation (r_w_ = 0.30), and small in meaningfulness for intrinsic motivation (r_w_ = 0.20), identified regulation (r_w_ = 0.26), external regulation (r_w_ = 0.27), amotivation (r_w_ = 0.10), and effort (r_w_ = 0.19).

All of the significant performance-approach correlations were positive in direction. The correlations were medium in meaningfulness with extrinsic motivation (r_w_ = 0.39), introjected regulation (r_w_ = 0.32), and external regulation (r_w_ = 0.32), and small with intrinsic motivation (r_w_ = 0.26), identified regulation (r_w_ = 0.26), amotivation (r_w_ = 0.19), positive affect (r_w_ = 0.18), effort (r_w_ = 0.20), and all forms of physical activity reporting (intent r_w_ = 0.17, objective r_w_ = 0.11, self-reported r_w_ = 0.25).

Last for the performance-avoidance goal, all the significant correlations all were positive in direction. The correlation were medium in meaningfulness for extrinsic motivation (r_w_ = 0.34) and introjected regulation (r_w_ = 0.31), and small for intrinsic motivation (r_w_ = 0.19), identified regulation (r_w_ = 0.23), external regulation (r_w_ = 0.28), amotivation (r_w_ = 0.14), and effort (r_w_ = 0.13). The correlation with self-reported physical activity was significantly different than zero thought the meta-analyzed correlate was less than small (r_w_ = 0.08) in meaningfulness.

Concerning the publication bias statistics, the statistics supported the initial meta-analyzed results. The fail-safe N values in all but two cases (mastery-approach and negative affect, and performance-avoidance and self-reported physical activity) were substantially larger than the number of samples. The trim and fill statistics also supported the robustness of the random effects correlations as minor differences arose. From the categorization for meaningfulness, the changes were a result of boarder line values between meaningfulness interpretation categories. The specific changes in interpretation were mastery-approach with intrinsic motivation from medium to large and with relative autonomy from large to medium, the performance-approach goal with identified regulation from small to medium and external regulation from medium to small, and the performance-avoidance goal and self-reported physical activity from negligible to small.

### 3.8. Result Summary

Six hypotheses, some with multiple comparisons, were tested. The support for each is summarized in Table 5. Overall, support was found for some aspect of each hypothesis. Issues arose with inconsistencies between the scale types when examining context differences. Additionally, we found little support for performance-avoidance differences, but we found support with extending our hypotheses to the mastery-avoidance goal. Many of the outcome correlate results were in line with our hypotheses.

## 4. Discussion

The purpose of this investigation was to use meta-analytic techniques to summarize the state of the 2 × 2 achievement goals across the sport and exercise psychology research literature. To do so, we examined a number of research questions ranging from the overall pattern of 2 × 2 achievement goal endorsement to the relationships of outcome correlates from samples collected in 22 different countries. Essentially, this quantitative review, along with past 2 × 2 achievement goal meta-analytic reviews [3,4], provide a full picture of Elliot’s [2,10] Hierarchical Model of Approach and Avoidance Motivation in the sport and exercise psychology literature.

For our mean level research questions, the results with sufficient sample size were examined for the two most common response scale types, 7-point or 5-point. Our first research question was fully supported. Across all studies and samples worldwide, the mastery-approach goal is endorsed significantly and very meaningfully, more than the other achievement goals. It is concerning that the pattern of goal endorsement after the mastery-approach goal differs by response scale type. The patterns differed once again when examining publication bias statistics (i.e., trim and fill) with mean level adjustments. For both the response scale samples, the mastery-avoidance and performance-approach goals were endorsed with overlapping 95% CI. This pattern was widened with the publication bias analyses. Surprisingly, the pattern of results with the 5-point response scale and the trim and fill statistics, placed the performance-avoidance goal above the performance-approach goal with both below the mastery-avoidance goal. The lowest performance-approach value within the 5-point scale types came from the Riou and colleagues [110] test of their French achievement goal questionnaire worded as the AGQ-S with a sample of exercisers. The notion that the performance-avoidance goal might be, or should be, endorsed to a greater extent than the performance-approach goal requires explanation. However, the main concern is the pattern of the achievement goal differing by response scale type.

The issue of differing levels of support by response scale type, appeared within our research question regarding patterns of responses by study context. For the remaining research questions, most samples were within the 7-point response scale option. Although, the traditional statistical support was not consistent with respect to the performance-avoidance goal and study context, the participants in PE endorsed the performance-avoidance goal more than those in sport and PA for the 7-point scale response studies. As worldwide statistics indicate, children and youth engagement in recommended daily amounts of physical activity are extremely low [143]. Across all children and youth, physical education is the main avenue in promoting and building lifelong physical activity skills and motivations. As found in the outcome correlate analyses, the performance-avoidance goal correlates are small to negligible. However, the small in magnitude correlations are not helpful in lifelong physical activity pursuits. Sport participants endorsed approach goals more than participants in both the PA and PE. The approach goals, both small in meaningfulness, were positively correlated with physical activity correlates. Physical activity correlate research [144] places team sports as a positive correlate with increased moderate-to-vigorous activity minutes. Although children and adolescents generally drop out of competitive sports before adulthood, sports are played across one’s life and one can come back to sports in adulthood. For instance, large scale research is encouraging in many regions of Europe concerning adult health-enhancing daily physical activity [145]. Sport participation seems to be a main avenue for meeting daily physical activity recommendations. Hence, positive youth sport experiences seem imperative in making headway with the physical activity epidemic.

Our third research question concerned all female samples endorsing the avoidance goals more than all male samples. Elliot [10] formed his thoughts based on historic research and thoughts that painted females compared to males as more failure anxious. Stein and Bailey [146], in their review of the literature, placed female anxiety in relation to failure on sex role socialization and child rearing practices. Throughout our analyses, we extended Elliot’s [10] performance-avoidance hypotheses to the mastery-avoidance goal. Our analyses supported all female samples endorsing the mastery-avoidance goal more than all male samples. Certainly, an understandable criticism of our hypothesis testing is not examining the samples with both males and females. It is unknown the potential of mixed samples completing a 2 × 2 achievement goal questionnaire together that is in the same room as routinely done with survey research. Of more concern is the unknown reason why mastery, and not the performance-avoidance goal difference, resulted. Lochbaum and colleagues [9] reported small yet statistically different than zero meta-analyzed correlations between both avoidance goals and gender. In the present review, male only samples did endorse the performance-approach goal more than female only samples in the same magnitude as the female only samples endorsed the mastery-avoidance goal more than male only samples. It could be that goal contrast scores (performance-approach–performance-avoidance, mastery-approach–mastery-avoidance) would hold gender specific differences.

The writing about and study of individualism and collectivism has a long history. Oyserman and colleagues [20] suggested individualism was first used in regard to the French Revolution, as detailed first by Burke in 1790 [147]. Since the French Revolution, a great deal of research has focused on contrasting individualism and collectivism [20]. We added to the large body of literature by examining whether more individualistic countries endorsed the mastery-approach goal more, and the performance-approach goal less, than more collectivist countries. We controlled for socioeconomic status. Support was found for the more individualistic countries endorsing the mastery-approach goal with, and without, socioeconomic status being controlled, but not for the performance-approach goal being endorsed less (i.e., a significant or at least small in meaningfulness negative correlation). Our result followed the sport-based dichotomous achievement goal findings regarding support for task orientation and no support for the ego orientation. It certainly could be that, in a globally competitive world, thoughts of beating others and performing well override individualism, or equal to individualism with a focus on personal improvement.

Our next research question also included individualism and collectivism with socioeconomic status based on Elliot’s [10] writings. Economic disadvantages have been linked to motivation variables for decades, such as the work by Zigler, Abelson, and Seitz [148]. As with our third and fourth hypothesis, we found support for the mastery-avoidance goal being endorsed by lower socioeconomic and interdependent countries, than higher socioeconomic and independent countries, but not for Elliot’s [10] initial thoughts concerning the performance-avoidance goal. Our knowledge of participant level socioeconomic status certainly was unknown, as was the knowledge of each participant’s thoughts on individualism and collectivism. However, again, the hypotheses concerning the avoidance goals have been consistent with mastery-avoidance goal support. Although, there can be advantages to a mastery-avoidance goal, such as studying game film for football to be sure not to do worse than a past football performance, it is hard to conjure up the benefits of such a mindset for repeated performances. Indeed, Lochbaum and Gottardy [3] reported a negative impact on performance.

With respect to our final research question, we examined a number of achievement motivation outcome correlates. Overall, our results followed past correlate meta-analyses on the dichotomous achievement goals [9], the approach-avoidance goals [3], and motivation climate [149]. These correlate analyses also pull together Lochbaum and colleagues’ [4] antecedent meta-analysis on the 2 × 2 achievement goals. Most certainly, the correlation pattern with the mastery-approach goal verifies the importance of promoting this goal, as well as a mastery motivation climate as both can be classified medium to large in meaningfulness related to valued achievement context outcomes, such as more intrinsically focused motivation and regulations, positive affect, and effort. Although small in magnitude, the meta-analyzed correlations with physical activity all place the mastery-approach goal as important to reducing the physical inactivity epidemic [143,145]. However, a note of caution is warranted. The mastery-approach goal is positively related with external regulation and extrinsic motivation. These relationships arecontrary to the mastery goal and climate frameworks. Research in education also points to the mastery-approach goal being linked to performance goal characteristics, such as social comparison [150]. In short, the mastery-approach goal in this, and past reviews, is associated with many positive achievement behaviors, thoughts, and emotions. It is also linked to less desirable and hypothesized constructs.

It is important to note that the remaining achievement goals are similar in magnitude and direction with the outcome correlates. For instance, all the goals are generally positively related to intrinsic motivation, positive affect, and effort. It is important to note the positive relationships with thoughts of amotivation and negative emotions between the performance-approach and both avoidance goals. More importantly is the similarity of results for both avoidance goals and the performance-approach goal, which suggest that these goals do not provide distinct information with the analyzed outcome correlates. This is certainly a theoretical and practical concern. The value of the avoidance goals seems to be within the goal contrast and performance literature. As meta-analyzed by Lochbaum and Gottardy [3], the performance goal contrast score, as found in a few studies [118], is medium in magnitude in relation to performance. That results seems unread or ignored in the literature to date.

### Limitations and Future Directions

Although this is the first meta-analysis, in sport and exercise psychology, to extensively examine a number of Elliot’s [10] hypotheses, potential context differences, outcome correlates, and essentially pull together a fuller picture of all 2 × 2 achievement goal works, a few limitations exist. As discussed by Biddle and colleagues [151], the study of achievement goal orientations is nearly always Category C evidence, defined as uncontrolled or non-random dominant trials. Thus, the overall impact on policy-makers is limited with respect to goal orientation research. The most apparent and pervasive policy-making impact is the importance of promoting the mastery-approach goal, mastery climate, and task orientation in youth sport and PE programs, given the consistent positive relation to valued constructs such as intrinsic motivation, effort, and positive affect. Another limitation is the unknown reason or reasons for the differing patterns in some instances of results, based on the response scale. Somewhat analogous to the present limitation is the one raised by Lochbaum and his colleagues [8,9] when results differed by the dichotomous achievement goal scale. Inconsistency in results by scale or response scale only adds confusion to the achievement goal literature. Testing of socioeconomic status and culture were unique. However, our analyses were limited, as knowing each participant’s socioeconomic status and thoughts of cultural individualism, is unknown. For instance, most likely, college student samples were not representative of their country as college students tend to be higher in socioeconomic status, that is positively correlated with higher individualism and lower collectivism [20]. This was true overall in the present data set. Last, it could be that additional published manuscripts in other languages were not found as the only articles in Spanish, and one each in Italian and Turkish were located. In the landscape of achievement goal meta-analytic reviews, only Lochbaum and his colleagues include articles in languages other than English. Researchers are encouraged to include all languages.

Even with the mentioned limitations, the present review greatly advanced the 2 × 2 achievement goal literature. Based on the findings and limitations, a few lines standout for future research. First, a consistent pattern has emerged, based on all approach-avoidance meta-analyses in that the relative importance of the avoidance goals seems limited. The meaningfulness is small to negligible in the antecedent [4,5] or outcome relationships [3,6], and in the present meta-analysis. Additionally, the endorsement of the avoidance goal is far less than that of the mastery-approach goal. Although, certainly understudied, one value of the avoidance goals seem to be within the goal contrast score, as reported in the Lochbaum and Gottardy [3] approach-avoidance and performance meta-analysis. Since the publication of Lochbaum and Gottardy’s meta-analysis [3], Lochbaum and Smith [95] and Lochbaum [91] demonstrated the importance of, not only the performance goal contrast score, but the mastery goal contrast score. It seems imperative that future research addresses the value of the avoidance goals to achievement driven thoughts, emotions, and behaviors. Along this line, whether the current set of 2 × 2 measures appropriately measures all the goals is unknown as the sport, PE, and PA specific measures never underwent revision, as did the education AGQ [11] to the AGQ-R [16]. Additionally, as mentioned in the introduction, the 3 × 2 model and measure, as well as the most recent 2 × 2 standpoints models, are theoretically improvements within the achievement goal literature. Given the overall similar pattern of the mastery-avoidance and both performance goals with the outcome correlates, it may be researchers in the psychology of sport, exercise, and physical education contexts who need to determine whether the current set of 2 × 2 measures need revision or even a different conceptual direction. One study or a series of studies would be a large task, or a task requiring new, sequential, well-planned investigations. Additionally, a number of advanced statistical procedures have been applied to achievement goal research and are worth considering [152,153]. Last, understanding the impact of socioeconomic status, culture, and gender require large and representative samples. Such an undertaking is worth research.

## 5. Conclusions

This meta-analytic summary provided a number of important findings regarding the state of Elliot’s and colleagues 2 × 2 achievement goals, as well as helping complete a picture of Elliot’s Hierarchical Model of Approach and Avoidance Motivation in the psychology of sport, PE, and PA domains. Hence, taken together with past meta-analyses on these goals, the present meta-analysis is important in shaping future research. Worth considering is whether sport and exercise psychology as a whole needs to move away from the 2 × 2 achievement goals, examine the goal contrast scores, attempt to revise the AGQ-S, PE, and PA, move to other models, or simply stay with the much-researched dichotomous goals. Regardless of the direction, what is known is that the mastery-approach is globally endorsed and related to valued achievement thoughts, emotions, and behaviors, and the other goals are minimal in association to detrimental achievement thoughts, emotions, and behaviors. Thus, if in doubt, promote the mastery-approach goal.

## Figures and Tables

**Table 1:** Summary information for included studies.

Ref #	Authors	Year	Country	Context	Activity	Goals	N	Sex	*M* Age	Data
[30]	Adie et al.	2008	UK	S	Team Mix	all	424	B	24.25	ML, r
[31]	Adie et al.	2010	UK	S	Soccer	all	91	M	13.82	ML, r
[32]	Agbuga et al.	2015	Turkey	PE	PE	all	221	B	16.04	ML
[33]	Baena-Extremera and Granero-Gallegos	2015	**Spain**	PE	PE	all	410	B	15.14	ML
[34]	Baena-Extremera et al.	2016	CR/Mexico/Spain	PE	PE	all	1811	B	12.49	ML
[35]	Barkoukis et al.	2011	Greece	S	Mix	all	1075	B	22.97	ML
[36]	Barkoukis et al.	2012	Greece	S	Basketball	all	221	M	23.71	ML
[37]	Bois et al.	2009	France	S	Golf	all	41	M	28.80	ML
[38]	Bono and Livi	2016	**Italy**	S	Swimming	all	96	B	NR	r
[39]	Castillo et al.	2011	**Spain**	S	Soccer	all	370	B	14.77	ML
[40]	Cecchini and Méndez-Giménez	2017	Spain	PA	Pre-service PE	all	408	B	20.15	ML, r
[41]	Çepikkurt and Yazgan İnanç	2012	**Turkey**	S	Handball	all	143	M	21.75	ML
[42]	Cetinkalp	2012	Turkey	S	Team Mix	all	208	B	16.35	ML
[43]	Chalabaev et al.	2008	France	S	Soccer	P	51	F	20.30	ML
[44]	Chen et al.	2009	Taiwan	PE	CAC	all	691	B	20.17	ML
[45]	Conroy et al.	2008	USA	S	Athletics	all	71	F	19.60	ML
[46]	Conroy and Elliot	2004	USA	PA	CAC	all	356	B	21.57	ML
[13]	Conroy et al.	2003	USA	PA	CAC	all	255	B	21.57	ML
[47]	Corrion et al.	2010	France	PE	PE	all	477	B	13.60	ML
[48]	Corrion et al.	2018	France	S	Ultra-trailers	all	221	B	43.00	ML
[49]	Cuevas et al.	2012	Spain	PE	PE	all	169	B	15.51	ML
[50]	Cuevas et al.	2013	**Spain**	PE	PE	all	390	B	15.41	ML
[51]	Cuevas et al.	2014	**Spain**	PA	PE	all	270	B	15.66	ML, r
[52]	Duff-Riddell and Louw	2011	South Africa	S	Horse Riders	all	83	F	13.82	ML
[53]	Ersöz et al.	2015	**Turkey**	S	Mix	all	820	B	21.37	ML
[54]	Fernández-Rio et al.	2014	Spain	S	Swimmers	all	19	B	17.10	ML
[55]	Fernández-Rio et al.	2017	Spain	S	Team Mix	all	48	F	25.14	ML
[56]	Fernández-Rio et al.	2018	Spain	S	Kayaking	all	3	NR	25.00	ML, r
[57]	Gao et al.	2011	USA	PE	PE	all	194	B	12.40	ML, r
[58]	Gao et al.	2013	USA	PE	PE	all	276	B	13.34	ML
[59]	Gardner et al.	2017	Australia	S	Mix	all	327	B	13.03	ML
[60]	Gardner et al.	2018	Australia	S	Mix	all	247	B	13.03	ML
[61]	Garn and Cothran	2009	USA	PA	CAC	all	224	B	19.44	ML
[62]	Garn and Sun	2009	USA	PE	PE	all	214	B	***13.00***	ML, r
[63]	Garn et al.	2011	USA	PE	PE	all	105	B	15.18	ML
[64]	Girad et al.	2019	Canada	PE	PE	Map, P	843	B	13.87	ML, r
[65]	Gómez-López et al.	2014	Spain	PE	PE	all	846	B	15.47	ML
[66]	Gonzalez-Cutre Coll et al.	2011	Spain	PE	PE	M, pap	46	B	13.39	ML
[67]	Gözmen Elmas & Aşçi	2017	**Turkey**	S	Mix	all	209	B	21.45	ML
[68]	Gråstén & Forsman	2018	Finland	S	Floorball	all	283	M	11.49	ML, r
[69]	Guan et al. (S1)	2007	USA	PE	PE	all	180	B	16.27	ML
	(S2)		USA	PE	PE	all	366	B	16.54	ML
[70]	Guan et al.	2013	USA	S	Team Mix	all	171	B	16.34	ML
[71]	Gucciardi	2010	Australia	S	Australian Football	all	214	M	16.80	ML
[72]	Gucciardi et al.	2012	Australia	S	Mix	all	423	B	25.64	ML
[73]	Gutiérrez et al.	2017	**Spain**	PE	PE	all	608	B	14.51	ML
[74]	Hagger et al. (S1)	2011	UK	PA	University	all	243	B	27.20	ML, r
	(S2)		Estonia	PA	University	all	216	B	23.40	ML
	(S3)		UK	PA	University	all	442	B	30.10	ML, r
[75]	Halvari et al.	2011	Norway	PE	PE	P	152	B	13.50	ML, r
[76]	Hsu et al. (S1)	2017	Taiwan	PE	PE	all	287	B	14.20	ML
	(S2)		Taiwan	PE	PE	all	296	B	14.10	ML
[77]	Hulleman et al.	2008	USA	S	Football	AP	237	M	***16.00***	ML, r
[78]	Isoard-Gautheur et al.	2013	France	S	Handball	all	309	B	15.40	ML
[79]	Isoard-Gautheur et al.	2016	France	S	Mix	all	360	B	21.00	ML
[80]	Jaakkola et al.	2016	Finland	S	Ice hockey	all	265	M	17.03	ML
[81]	Jackson et al.	2010	Australia	S	Mix	all	82	B	22.72	ML
[82]	Kaye et al.	2008	USA	PA	CAC	all	372	B	21.20	ML
[83]	Kaye et al.	2015	USA	S	Competitive	all	73	B	12.61	ML
[84]	Kazak	2018	Turkey	PA	LTPA	all	401	B	26.16	ML, r
[85]	Kesilmiş, & Yıldız	2018	Turkey	S	Athletics	all	70	B	***20.94***	ML
[86]	Koh and Wang	2015	Singapore	S	Mix	all	101	B	16.70	ML
[87]	Lench et al.	2010	USA	S	Dance	all	109	B	20.12	ML
[88]	Li	2010	Taiwan	S	Team Mix	all	645	B	16.60	ML
[89]	Li	2013	Taiwan	S	Handball	all	160	B	17.00	ML
[90]	Li et al.	2011	Taiwan	S	Handball	all	164	NR	15.70	ML
[91]	Lochbaum	2014	USA	S	Mix	all	65	B	***18.00***	ML
[92]	Lochbaum et al.	2016	Chile	S/PA	Team Mix/CAC	all	221	B	22.00	ML
[93]	Lochbaum, Litchfield et al.	2013	USA	PA	LTPA	all	213	B	37.21	ML, r
[94]	Lochbaum, Podlog et al.	2013	USA	PA	LTPA	all	804	B	20.88	ML
[95]	Lochbaum and Smith	2015	USA	S	CAC	all	175	B	***20.00***	ML
[96]	Lochbaum et al.	2009	USA	PA	CAC	all	286	B	***20.00***	ML
[97]	Méndez-Giménez et al.	2014	Spain	PE	PE	all	351	B	14.26	ML, r
[98]	Méndez-Giménez et al.	2015	Spain	PE	PE	all	295	B	14.20	ML, r
[99]	Méndez-Giménez et al.	2012	**Spain**	PE	PE	all	421	B	14.56	ML, r
[100]	Méndez-Giménez et al.	2013	**Spain**	PE	PE	all	359	B	15.67	ML, r
[101]	Méndez-Giménez et al.	2015	**Spain**	PE	PE	all	385	B	14.25	ML
[102]	Moreno et al.	2010	Spain	PA	LTPA	all	727	B	32.57	ML
[103]	Morris and Kavussanu	2008	UK	S	Team Mix	all	230	B	20.30	ML
[104]	Moreno Murcia et al.	2008	Spain	PA	LTPA	all	727	B	32.57	ML
[105]	Nien and Duda	2008	UK	S	Mix	all	446	M	22.17	ML
[106]	Ntoumanis et al.	2009	UK	S	Darts	all	138	B	19.30	ML, r
[107]	Ortiz-Camacho et al.	2017	Spain	PE	PE	all	2002	B	14.99	r
[108]	Partridge et al.	2014	USA	S	Crossfit	all	144	B	34.40	ML
[109]	Puente-Diaz	2013	Mexico	S	Tennis	all	204	B	14.13	ML
[110]	Riou et al. (S1)	2012	France	S	Mix	all	270	B	***23.30***	ML
	(S2)		France	PA	LTPA	all	234	B	***73.20***	ML
	(S3)		France	PE	PE	all	255	B	***15.50***	ML
[111]	Ruiz-Juan & Baena-Extremera	2015	CR/Mexico/Spain	PA	PE	all	2168	B	12.49	ML, r
[112]	Sáenz-López et al.	2017	**Spain**	S	Basketball	all	57	F	13.02	ML
[113]	Schantz and Conroy	2009	USA	S	Golf	all	25	B	19.60	ML
[114]	Skjesol and Halvari	2005	Norway	PA	LTPA	P	231	B	16.70	ML, r
[115]	Spray and Warburton	2011	UK	PE	PE	map, P	432	B	13.18	ML
[116]	Spray et al.	2013	UK	PE	PE	map, P	866	B	11.29	ML
[117]	Stenling et al.	2014	Sweden/Australia	S	Team Mix	all	315	B	19.98	ML
[15]	Stevenson and Lochbaum (S1)	2008	USA	PA	CAC	all	386	B	20.00	ML
	(S2)		USA	PA	CAC	all	148	B	20.00	ML
[118]	Stoeber and Crombie	2010	UK	S	Athletics	all	192	B	20.70	ML
[119]	Stoeber, Stoll, et al.	2009	Finland	S	Ice Hockey	all	138	M	14.50	ML
[120]	Stoeber, Uphill, et al. (S1)	2009	UK	S	Triathlon	all	112	B	36.50	ML
	(S2)		UK	S	Triathlon	all	339	B	37.20	ML
[121]	Su et al.	2015	USA	PA	CAC	all	361	B	19.97	ML
[122]	Theodosiou et al.	2018	Greece	S	Tennis	all	226	B	15.21	r
[123]	Trenz and Zusho	2011	USA	S	Swimming	all	119	B	14.76	r
[124]	Turner et al.	2012	UK	S	Netball	all	21	F	21.09	ML
[125]	Turner et al.	2013	UK	S	Cricket	all	42	M	16.45	ML
[126]	Vallerand et al.	2008	Canada	S	Mix	P	67	B	16.10	ML
[127]	Vansteenkiste et al.	2010	Belgium	S	Soccer	pap	304	M	24.66	ML, r
[128]	Verner-Filion et al.	2017	Canada	S	Ice Hockey	P	598	M	16.56	ML, r
[14]	Wang et al.	2007	Singapore	PE	PE	all	647	B	13.92	ML, r
[129]	Wang, Koh, et al.	2009	Singapore	S	Basketball	map, P	264	B	15.68	ML, r
[130]	Wang et al.	2008	Singapore	PE	PE	all	493	B	14.32	ML
[131]	Wang et al.	2010	Singapore	PE	PE	all	781	B	15.24	ML
[132]	Wang et al.	2009	USA	PA	CAC	all	309	B	21.37	ML
[133]	Wang et al.	2016	Singapore	PE	PE	all	1810	B	***16.00***	ML, r
[134]	Wang et al.	2011	Singapore	S	Mix	all	374	B	14.50	ML
[135]	Warburton	2017	UK	PE	PE	all	655	B	12.74	ML
[136]	Warburton and Spray	2008	UK	PE	PE	all	140	B	11.37	ML
[137]	Warburton and Spray	2009	UK	PE	PE	all	511	B	13.18	ML
[138]	Warburton and Spray	2013	UK	PE	PE	all	301	B	13.16	ML
[139]	Weltevreden et al.	2018	The Netherlands	S	Team Mix	all	140	B	15.50	ML
[140]	Yeatts and Lochbaum	2013	USA	PA/S	Mix	all	258	B	20.46	ML
[141]	Zarghimi et al.	2010	Iran	S	Mix	all	184	B	23.25	ML
[142]	Zhang et al.	2016	USA	PA	CAC	all	325	B	21.40	ML

Note. (S1) (S2) and (S3) indicate distinct samples. A country in **bold** indicates the language of the written article. An age in ***bold italics*** indicates estimated. UK = United Kingdom; USA = United States of America; CR = Costa Rica; S = sport; PE = physical education; PA = physical activity; P = both performance goals; LTPA = leisure time physical activity; CAC = college activity classes; NR = not reported and cannot be estimated or inferred; M = male only sample; B = both males and female sample; F = female only sample; All = all four achievement goals included; ML = study provided mean level data; r = study provided correlation data.

**Table 2:** Random effects model results and publication bias statistics for estimated means for all goals for two scale types.

	Effect Size Statistics				Publication Bias Statistics			
Variables	*k*	*M (SE)*	95% CI	*I* ^2^	Missing Studies	N Fill, Direction	*M*	95% CI
1-7 Scale								
MAp	106	5.66 (0.07)	5.53, 5.80	H	283,374.4	17, left	5.51	5.38, 5.65
MAv	103	4.45 (0.07)	4.32, 4.58	H	86,943.4	11, left	4.33	4.19, 4.46
PAp	114	4.48 (0.08)	4.33, 4.64	H	99,402.3	21, right	4.71	4.55, 4.89
PAv	111	4.15 (0.07)	4.02, 4.28	H	80,656.3	---	---	---, ---
1-5 Scale								
MAp	28	4.02 (0.09)	3.85, 4.19	H	84,587	---	---	---, ---
MAv	27	3.33 (0.10)	3.14, 3.52	H	33,346.7	2, right	3.40	3.20, 3.61
PAp	28	3.22 (0.10)	3.02, 3.42	H	32,107.8	6, left	3.04	2.81, 3.27
PAv	27	3.12 (0.09)	2.95, 3.29	H	26,545.1	6, right	3.27	3.10, 3.44

Note. MAp = mastery approach goal; MAv = mastery avoidance goal; PAp = performance approach goal; PAv = performance avoidance goal; H = high heterogeneity; *k* = number of samples; *M* = random effects estimated mean; *SE* = standard error; CI = confidence interval.

**Table 3:** Group mixed model results for the context moderator variable for both the 7- and 5-pt scales.

Scale	Goal	Category	*k*	*M (SE)*	95% *CI*	*Q*_b_	*p*
7-pt	MAp	PA	29	5.62 (0.09)	5.45, 5.79		
		PE	23	5.29 (0.16)	4.97, 5.61		
		S	39	5.86 (0.01)	5.84, 5.88	7.34	<0.05
5-pt		PA	4	3.58 (0.17)	3.25, 3.91		
		PE	11	3.79 (0.12)	3.56, 4.03		
		S	5	4.37 (0.15)	4.07, 4.66	13.97	<0.001
7-pt	MAv	PA	27	4.25 (0.13)	3.99, 4.51		
		PE	23	4.41 (0.15)	4.12, 4.69		
		S	38	4.54 (0.10)	4.33, 4.74	2.94	0.23
5-pt		PA	4	2.99 (0.11)	2.78, 3.21		
		PE	11	3.31 (0.12)	3.07, 3.55		
		S	5	3.51 (0.16)	3.19, 3.83	7.73	<0.05
7-pt	PAp	PA	30	4.36 (0.14)	4.09, 4.63		
		PE	25	4.39 (0.16)	4.07, 4.71		
		S	43	4.56 (0.13)	4.32, 4.81	1.37	0.50
5-pt		PA	7	3.10 (0.09)	2.93, 3.28		
		PE	11	3.29 (0.17)	2.96, 3.62		
		S	5	3.45 (0.10)	3.26, 3.65	6.89	<0.05
7-pt	PAv	PA	30	4.09 (0.13)	3.84, 4.34		
		PE	24	4.56 (0.15)	4.26, 4.85		
		Sport	41	3.93 (0.12)	3.70, 4.16	10.98	<0.01
5-pt		PA	7	2.95 (0.10)	2.76, 3.14		
		PE	11	3.11 (0.16)	2.79, 3.43		
		Sport	4	3.31 (0.19)	2.93, 3.69	3.06	0.22

Note. MAp = mastery approach goal; MAv = mastery avoidance goal; PAp = performance approach goal; PAv = performance avoidance goal; H = high heterogeneity; *k* = number of samples; *M* = random effects estimated mean; *SE* = standard error; CI = confidence interval.

**Table 4:** Random effects size statistics and publication bias statistics for each correlate category by approach-avoidance goal.

		Effect Size Statistics					Publication Bias Statistics			
Correlates	Goal	*k*	*r_w_*	95% CI	*Z* Value	*p* Value	Fail-Safe N	N Fill	Direction	*r_c_*
Relative Autonomy	MAp	6	0.51	0.40, 0.60	7.86	0.00	1345	1	left	0.47
	MAv	6	0.14	−0.04, 0.31	1.53	0.13				
	PAp	6	0.11	−0.02, 0.24	1.59	0.11				
	PAv	6	−0.05	−0.21, 0.12	−0.56	0.58				
Extrinsic Motivation	MAp	5	0.36	0.25, 0.46	6.04	0.00	563	0		
	MAv	5	0.33	0.26, 0.40	8.52	0.00	471	0		
	PAp	5	0.39	0.28, 0.50	6.18	0.00	704	1	right	0.41
	PAv	5	0.34	0.24, 0.43	6.26	0.00	472	0		
Intrinsic Motivation	MAp	19	0.48	0.42, 0.54	12.86	0.00	9246	5	right	0.52
	MAv	19	0.20	0.13, 0.26	6.12	0.00	1538	1	right	0.20
	PAp	19	0.26	0.20, 0.33	8.00	0.00	2550	0		
	PAv	19	0.19	0.13, 0.26	5.47	0.00	1402	1	right	0.19
Identified Regulation	MAp	11	0.42	0.32, 0.52	7.29	0.00	2489	0		
	MAv	11	0.26	0.18, 0.34	6.03	0.00	921	1	right	0.26
	PAp	11	0.26	0.19, 0.33	6.87	0.00	857	4	right	0.30
	PAv	11	0.23	0.13, 0.32	4.60	0.00	673	2	right	0.26
Introjected Regulation	MAp	10	0.28	0.18, 0.38	5.10	0.00	1078	0		
	MAv	10	0.30	0.22, 0.37	7.43	0.00	1149	0		
	PAp	10	0.32	0.24, 0.40	7.56	0.00	1337	1	right	0.33
	PAv	10	0.31	0.23, 0.38	7.62	0.00	1190	0		
External Regulation	MAp	12	0.15	0.07, 0.22	3.79	0.00	322	0		
	MAv	12	0.27	0.21, 0.33	8.67	0.00	1131	4	left	0.22
	PAp	12	0.32	0.26, 0.37	9.90	0.00	1617	2	left	0.29
	PAv	12	0.28	0.23, 0.33	10.22	0.00	1199	0		
Amotivation	MAp	13	−0.22	−0.40, −0.02	−2.12	0.03	906	2	left	−0.26
	MAv	13	0.10	0.01, 0.19	2.26	0.02	230	0		
	PAp	13	0.19	0.11, 0.26	4.82	0.00	734	1	left	0.18
	PAv	13	0.14	0.09, 0.20	5.08	0.00	398	2	left	0.13
Negative Affect	MAp	3	−0.12	−0.18, −0.05	−3.59	0.00	5	0		
	MAv	3	0.12	−0.13, 0.36	0.96	0.34				
	PAp	4	0.03	−0.10, 0.15	0.44	0.66				
	PAv	3	0.10	−0.06, 0.26	1.25	0.21				
Positive Affect	MAp	4	0.42	0.37, 0.46	15.63	0.00	224	1	right	0.42
	MAv	4	0.12	−0.06, 0.30	1.33	0.18				
	PAp	5	0.18	0.06, 0.31	2.84	0.00	69	0		
	PAv	4	0.04	−0.13, 0.21	0.49	0.63				
Effort	MAp	7	0.40	0.22, 0.55	4.13	0.00	952	0		
	MAv	8	0.19	0.09, 0.28	3.76	0.00	190	0		
	PAp	10	0.20	0.10, 0.29	3.92	0.00	292	0		
	PAv	9	0.13	0.04, 0.23	2.71	0.01	110	1	right	0.16
Intent PA	MAp	5	0.38	0.31, 0.45	10.27	0.00	580	0		
	MAv	5	0.17	0.12, 0.22	6.39	0.00	105	3	right	0.21
	PAp	5	0.19	0.10, 0.27	4.38	0.00	135	1	right	0.21
	PAv	5	0.06	−0.03, 0.16	1.33	0.18				
Objective PA	MAp	2	0.23	0.14, 0.33	4.73	0.00		0		
	MAv	2	0.00	−0.10, 0.10	−0.02	0.99				
	PAp	2	0.11	0.01, 0.21	2.16	0.03		0		
	PAv	2	0.09	−0.01, 0.19	1.77	0.08				
Self-reported PA	MAp	6	0.31	0.19, 0.43	4.81	0.00	467	2	right	0.39
	MAv	6	0.05	−0.04, 0.15	1.09	0.27				
	PAp	7	0.25	0.19, 0.30	7.75	0.00	354	0		
	PAv	7	0.08	0.04, 0.12	4.09	0.00	33	2	right	0.10

Note. MAp = mastery-approach goal; MAv = mastery-avoidance goal; PAp = performance-approach goal; PAv = performance-avoidance goal; *k* = number of samples; *r_w_* = random effects correlation; *r_c_* = trim and fill random effects correlation.

**Table 5:** Summary of examined research questions.

Research Questions		Summary
1	The mastery-approach goal would be endorsed more than the other goals.	Supported
2	Participants in PE classes would endorse the avoidance goals more than individuals competing in sports and in PA.	Limited/inconsistent support
	Participants in sports would endorse the performance approach goal more than those in the PA and PE groups and the mastery approach goal more than the PE students.	Supported
	Participants in sports would endorse the mastery approach goal more than the PE students.	Supported
3	Females would endorse the performance-avoidance more than males.	Not supported
	Females would endorse the mastery-avoidance goal more than males.	Supported
	Males would endorse the performance-approach goal more than females.	Supported
4	Countries more culturally individualistic (independent) would endorse the mastery-approach goal more than countries less individualistic (more interdependent).	Supported
	Countries more culturally individualistic (independent) would endorse the performance-approach goal less than countries less individualistic (more interdependent).	Not supported
5	Samples from lower socioeconomic and interdependent countries from lower overall incomes would endorse the performance-avoidance goal more than higher socioeconomic and independent countries.	Not supported
	Samples from lower socioeconomic and interdependent countries from lower overall incomes would endorse the mastery-avoidance goal more than higher socioeconomic and independent countries.	Supported
6	The mastery-approach goal would be related positively to correlates such as RAI, intrinsic motivation, positive affect, effort, PA and negatively with amotivation and negative affect.	Supported.
	The performance-approach goal pattern would be related positively with the correlates mentioned above with the mastery-approach goal but lower in magnitude.	Supported.
	The avoidance goal correlate patterns would be small to negligible in magnitude.	Supported.

## Supplementary Materials

The following are available online at https://www.mdpi.com/2254-9625/10/1/15/s1, Table: PRISMA example of full electronic search strategy.
