# Supplementary material for: The 2 × 2 Achievement Goals in Sport and Physical Activity Contexts: A Meta-Analytic Test of Context, Gender, Culture, and Socioeconomic Status Differences and Analysis of Motivations, Regulations, Affect, Effort, and Physical Activity Correlates
Source: Eur J Investig Health Psychol Educ. 2019 Nov 8;10(1):173–205. doi: 10.3390/ejihpe10010015 (PMC8314248; doi:10.3390/ejihpe10010015)
Supplement: Supplementary file 1 [file ejihpe-10-00015-s001.pdf]

Supplement. PRISMA example of full electronic search strategy

Steps

01. EBSCO as main database engine.
02. Selected SPORTDiscus, PsycINFO, and ERIC
03. Selected the ‘advanced search option’ to bring up three distinct boxes in which to type
04. Typed key terms in three distinct boxes
05. Box 1 mastery-approach goal
06. Box 2 sports
07. Box 3 approach and avoidance goal orientations
08. Limited search to a specific year for instance 1999
09. Limited search to ‘journal articles’ by checking this box
10. Changed box 1 to performance-approach goal
11. Changed box 1 to mastery-avoidance goal
12. Changed box 1 to performance-avoidance goal
13. Returned box 1 to mastery-approach goal
14. Changed box 2 to competitive sports
15. Changed box 2 to physical activity
16. Changed box 2 to recreation
17. Changed box 2 to leisure-time physical activity
18. Changed box 2 to physical education
19. Changed box 2 to PE
20. Changed box 2 to exercise
21. Returned box 1 to performance-approach goal
22. Redid steps 14-20
23. Returned box 1 to mastery-avoidance goal
24. Redid steps 14-20
25. Returned box 1 to performance-avoidance goal
26. Redid steps 14-20
27. Redid steps 10-26 with box 3 changed to achievement goal orientations
28. Redid steps 10-26 with box 3 changed to Elliot’s 2 x 2 goals
29. Started at step 04 for the next year, and so on until 2018
